# Muscle Antioxidant Activity and Meat Quality Are Altered by Supplementation of Astaxanthin in Broilers Exposed to High Temperature

**DOI:** 10.3390/antiox9111032

**Published:** 2020-10-23

**Authors:** Abdolreza Hosseindoust, Seung Min Oh, Han Seo Ko, Se Min Jeon, Sang Hoon Ha, Aera Jang, Ji Seon Son, Gur Yoo Kim, Hwan Ku Kang, Jin Soo Kim

**Affiliations:** 1 College of Animal Life Sciences, Kangwon National University, Chuncheon 24341, Korea; hosseindoust@kangwon.ac.kr (A.H.); Kofeed@kangwon.ac.kr (H.S.K.); tpals87@naver.com (S.M.J.); hosseindoust@asia.com (S.H.H.); gykim@kangwon.ac.kr (G.Y.K.); 2 Gyeongbuk Livestock Research Institute, Yeongju 63052, Korea; seungmin4@naver.com; 3 Department of Animal Products and Food Science, Kangwon National University, Chuncheon 24341, Korea; ajang@kangwon.ac.kr; 4 Poultry Devision, National Institute of Animal Science, Rural Development Administration, 321-11, Daegwallyeongmaru-gil, Daegwallyeong-myeon, Pyeongchang-gun, Gangwon-do 25342, Korea; wltjs1206@korea.kr

**Keywords:** antioxidant activity, malondialdehyde, malondialdehyde, superoxide dismutase, catalase, meat color, heat stress

## Abstract

This study investigated the effect of dietary astaxanthin (AST) on the meat quality, antioxidant status, and immune response of chickens exposed to heat stress. Four hundred and eighty male broilers were assigned to four treatments including AST0, AST20, AST40, and AST80 with 0, 20, 40, and 80 ppm astaxanthin supplementation levels, respectively. There was a linear decrease of malondialdehyde (MDA) in leg muscle. Catalase and superoxide dismutase levels in the plasma were linearly increased. There was a linear increase in the level of total antioxidant capacity in the leg muscle. The 3-ethylbenzothiazoline-6-sulfonate reducing activity of leg muscle was significantly increased in the AST80 treatment. The AST40 treatment showed an increase in 2,2-diphenyl-1-picrylhydrazyl radical scavenging capacity of leg muscles. Breast meat redness and yellowness were linearly increased. The astaxanthin-supplemented treatments exhibited lower drip loss and MDA concentration of leg muscle compared with the AST0 treatment at days 3 and 9 of storage. Supplementation of 40 or 80 mg/kg astaxanthin significantly decreased heat shock protein (HSP)27, HSP70, tumor necrosis factor alpha, and interleukin-6 expression in the livers. The feather corticosterone was significantly lower in the astaxanthin-supplemented treatments than in the AST0 treatment. In conclusion, astaxanthin decreased the hyperthermic stress level and improved meat quality, and antioxidant status of chickens exposed to heat stress.

## 1. Introduction

Environmental temperatures exceeding the thermo-neutral zone cause heat stress. The continuous selection of broiler chickens for fast growth has increased their vulnerability to heat stress than other farm animals. High ambient temperature in summer is thus a serious stressor for broiler chickens, resulting in poor health status, welfare, growth, and carcass quality [1,2,3,4,5]. In addition to decreased growth performance, exposure of broiler chicks to heat stress increases the oxidative reactions that compromise meat quality by affecting ultimate pH, water-holding capacity, meat color, and meat tenderness [4,6], resulting in lower marketability. Moreover, the high content of polyunsaturated fatty acids in chicken muscle can increase the susceptibility of meat to the oxidative deterioration [6]. Lipid oxidation occurs when there is an imbalance balance between the scavenging capacity of antioxidants and the generation of free radical [7,8,9]. Free radicals and reactive oxygen species damage several enzymes and macromolecules and can lead to severe oxidative injuries, with the potential of impacting the lipid oxidation and health status of animals. The body is naturally able to defend against intracellular oxidative damage by controlling oxidative materials and free radicals through an enzymatic antioxidant system. Several enzymes, including superoxide dismutase (SOD), catalase, and glutathione peroxidase (GPx), are able to counteract with free radicals and affect the meat and lipid peroxidation levels [7,10]. Under stress conditions, SOD and catalase have crucial roles as superoxide anion scavenger by decomposing hydrogen peroxides to superoxide anion and water [11]. Therefore, improving the oxidative status and oxidative stability of meat is correlative to the alleviation of heat stress detrimental influences.

The use of synthetic or natural antioxidants has recently received increasing attention in the poultry industry regarding their effects on oxidative stress and meat quality [1,12]. The beneficial effects of dietary antioxidants are much more highlighted during high ambient temperatures. Astaxanthin, a blood-red carotenoid, is derived from *Phaffia rhodozyma* yeast and is considered as an alternative to the other carotenoids [8]. The antioxidant capacity of astaxanthin is approximately ten times greater than those usual carotenoids including zeaxanthin, tunaxanthin, lutein, β-carotene, and cantaxanthin, and also a hundred times higher than vitamin E [13]. Apart from the aforementioned antioxidant capacity, astaxanthin can also be used as a pigment in aquaculture to increase the pink color of meat in salmonid [14] or rainbow trout [15]. To the broiler industry, meat and skin color are known as major quality issues for meat selection by consumers in the market [7,16,17]. The manipulation of meat color is a current concern to satisfy market preferences. To our knowledge, there has been a lack of comprehensive studies regarding the antioxidant roles of dietary astaxanthin in a dose-dependent manner not only on the meat quality but also on the immune system of broiler chickens during acute heat stress. Therefore, this study was aimed to determine the influences of supplementary astaxanthin on meat quality, antioxidant stability, and immune status in broilers under acute heat stress.

## 2. Materials and Methods

### 2.1. Experimental Design, Animal, Housing, and Diets

The experiment was approved by the Institutional Animal Care and Use Committee, Kangwon National University (ethical code: KW-170519-1). Four hundred and eighty male broilers (Ross^®^ 308, 36.46 ± 0.14 g, 1 day old; Aviagen Inc., Huntsville, AL, USA) were used for 35 days (2 phases; starter d 1 to 21, finisher d 22 to 35) feeding trials. Four treatments were designated according to astaxanthin supplementation (AST) levels (0, 20, 40, and 80 ppm). Astaxanthin was supplemented to the diets from the beginning of study (day 1). Each treatment has 8 replicates with 15 birds per pen (floor pen, w2300 × d1500 × h800 mm), where the rice hull was covered as litter. Temperature and humidity were controlled by an automatic ventilation system according to the Ross Broiler Management Handbook [18] during weeks 1, 2, and 3 the average temperature of broiler house was 32.2 °C, 30.2 °C, and 26.5 °C respectively. At the start of wk 4, the high temperature was applied to broiler chickens by rising up room temperature to 32.8 °C and 30.2 °C for 8 h (09:00–17:00) at weeks 4 and 5, respectively. Feed and water were provided ad libitum. The experimental diets were mash form and formulated to meet the nutrient requirements of Ross 308 Nutritional specification [18]. Astaxanthin was a powder type of supplement which was extracted from *Haematococcus* algae and containing 8% of xanthophyll, and premixed with corn and soybean meal before the main mixing then mixed using a vertical mixer for treatment diets.

### 2.2. Sample Collection

At the end of feeding trial (35 d), a total of 64 birds, 16 birds per treatment were subjected to the analysis for carcass characteristics, relative weights of organs, meat quality, antioxidant states, mRNA expression related to heat stress, and wing feather corticosterone evaluation. All birds were selected by similar body weight (BW) according to the average BW of a treatment. Feathers of all selected birds were collected from the left-wing of birds and covered by aluminum foil then stored at 25 °C temperature before analysis. After feather collection, blood samples were collected by the syringe from the wing vein. Collected blood was moved into non-treated vacuum tubes, and placed at 25 °C temperature for separation of plasma, then centrifuged at 2500× *g* for 10 min. Serum was aspirated and located in a 2.5 mL centrifuge tube then stored at −20 °C before analysis for malondialdehyde (MDA), GPx, SOD, myeloperoxidase (MPO), and total antioxidant capacity (TAC). All selected birds were decapitated at the first cervical vertebrae. After defeathering and removal of organs and feet, weights of the carcass, breast muscles, drumsticks, and abdominal fat were measured then stored at −20 °C for meat quality and antioxidant status analysis, and weights of liver, spleen, and bursa of Fabricius were measured then stored at −80 °C for gene expression analysis.

### 2.3. Meat Quality

Meat color of leg muscles was measured after slaughtered with a Chroma Meter CR-400 instrument (Minolta Co., Osaka, Japan) using International Commission on Illumination (CIE) L* (lightness), CIE a* (redness), and CIE b* (yellowness). Drip loss (%), cooking loss (%), shear force (%), pH, and MDA were analyzed during storage 1, 3, 6, and 9 days at 4 °C. Drip loss was measured using 2 g of meat sample placed in plastic containers where located stainless mesh inside [19]. For determining the cooking loss, 3 g of meat were heated in plastic bags separately in a water bath (85 °C) for 20 min, and cooled at room temperature then cooking loss was calculated as (sample weight before cookings/sample weight after cooking)/sample weight before cooking × 100 [20]. To measure shear force, a texture analyzer (TA-XT2i, Stable Microsystems, Surrey, UK) equipped with a Warner-Bratzler shear blade, a 25 kg load cell, and a test speed setting at 2.0 mm/s was used with the maximum force (kg) [21]. pH was measured as follows the minor modified method was described by Kim et al. [22]. In brief, 5 g of meat was homogenized with distilled water (45 mL) for 15 s using a homogenizer (DIAX 900, Heidolph, Kelheim, Germany), and the pH was determined using an Orion 230A pH meter (Thermo Fisher Scientific, Waltham, MA, USA). After pH determination, homogenized samples were filtered through Watman no. 2 (Hillsboro, OR, USA). l mL of filtered solution, serum and different 3 concentration levels of diluted MDA standard (Cayman Chemical, Ann Arbor, MI, USA) were moved into screw cap glass tubes, and 2 mL of TCA-TBA-HCl (trichloroacetic acid–thiobarbituric acid–hydrochloric acid) reagent were added all sample tubes. Tubes were caped, heated in boiling water 15 min and centrifuged at 1500 rpm for 10 min. The 250 µL of supernatant was allocated 96 well microplates then measured absorbance at 520 nm using a microplate reader (Power Wave XS, BIoTeK, Winooski, VT, USA). MDA concentration was determined using the MDA standard curve.

### 2.4. Antioxidant Enzyme Activity in Blood and Muscle

Antioxidant enzyme activity in muscle and serums was pre-treated and measured as following the method described by Cayman kit manual (Enzyme activity assay, Cayman Chemical, Ann Arbor, MI, USA). The muscles and serums were subjected for the assessment of the activity of SOD (Cat #706002, Cayman Chemical, Ann Arbor, MI, USA), glutathione (Cat #703002, Cayman, Cayman Chemical, Ann Arbor, MI, USA), and catalase (Cat #707002, Cayman Chemical, Ann Arbor, MI, USA). Microplate reader (Power Wave XS, BIoTeK, Winooski, VT, USA) was used for detecting the absorbance according to the Cayman kit’s manufacture manual.

### 2.5. Radical Scavenging Activity

The 2,2-diphenyl-1-picrylhydrazyl (DPPH) radical scavenging activity and 3-ethylbenzothiazoline-6-sulfonate (ABTS^+^) reducing activity were evaluated with the supernatant collected from thigh meat according to Blois [23]. Briefly, 200 μL of supernatant was placed in 5 mL centrifuge tube and added to both 800 μL deionized distilled water (DDW) and 1 mL methanolic DPPH solution (0.2 mM). The mixture was vortexed and kept at room temperature for 30 min. A tube containing 1 mL of methanolic DPPH solution (0.2 mM) and 1 mL of DDW was used for the control. The final mixtures were allocated into 96 well microplates then the absorbance of the solution was measured at 517 nm using a Microplate reader (Power Wave XS, BIoTeK, Winooski, VT, USA). The percentage of DPPH radical scavenging was determined from the following equation: DPPH radical scavenging activity (%) = (1 − (absorbance value of testing solution/absorbance value of control solution)) × 100. The determination of ABTS^+^ reducing activity and the preparation of the ABTS^+^ solution were conducted as following the method described by Erel [24]. The prepared ABTS solution was diluted with ethanol for adjusting absorbance approximately 0.70 at 734 by using a UV spectrophotometer (Optizen 3220UV, MECASYS, Daejeon, Korea). The diluted ABTS^+^ solution (3 mL) was mixed with 20 μL of supernatant was added to the diluted ABTS^+^ solution (3 mL) and the absorbance was measured by a UV spectrophotometer at 734 nm. The ethanol was used as a blank. The percentage inhibition was obtained by the following equation: ABTS^+^ reducing activity (%) = ((absorbance of the control − the absorbance of the sample)/absorbance of the control) × 100.

### 2.6. Hepatic Gene Expression

Total RNA was extracted from liver tissue using Trizol (Invitrogen, Carlsbad, CA, USA), and quantified using with the Thermo Scientific™ μDrop™ Plate (Thermo Scientific, Waltham, MA, USA) and Multiscan GO (Thermo Scientific, Waltham, MA, USA) at an absorbance ratio of 260/280. The 500 ng of total RNA was reverse transcribed to complementary DNA (cDNA) using Improm-II Reverse transcription system (Promega, Fitchburg, MA, USA). Polymerase chain reaction (PCR) condition and selection of primers were conducted following the description by Quinteiro-Filho et al. [25], Kang et al. [26], and Zoidis et al. [27]. Reverse transcription-quantitative real-time PCR (RT-qPCR) was performed using the Real-Time System (Mx3000P, Stratagen, La Jolla, CA, USA). The RT-qPCR primers including HSPs (HSP 27, HSP 40 and HSP70), TNF-α (tumor necrosis factor-alpha), IL-6, IL-1β, IL-13, GPx4, and β-actin were designed (Table 1). The PCR mixture consisted of 10 ul SYBR master mix (Cat #600882, Agilent Tech., Santa Clara, CA, USA), 0.25 uM of each gene-specific primer, and 2–5 µL of a 1:10 dilution of cDNA, and the final volume of the mixture was adjusted 20 µL adding diethyl phosphorocyanidate-treated water. The amplification conditions were as following: an enzyme activation step for 2 min at 95 °C, followed by denaturation and annealing/extension steps involving 40 cycles each for 5 s at 95 °C and then for 5 s at 60 °C (or each primer’s annealing temperature). The β-actin as a housekeeping gene was used for the normalizing of gene expression. The relative mRNA expression was calculated using 2^−ΔΔ^CT method.

### 2.7. Feather Corticosterone

The calamus of collected feathers was removed, and chopped finely by scissors then measured the weights then feather corticosterone was extracted by following the methanol extraction method [28]. Extracted feather corticosterone was analyzed using the ELISA kit (Corticosterone ELISA kit, Enzo life Sciences, Farmingdale, NY, USA). The feather corticosterone was expressed as pg/mg.

### 2.8. Statistical Analysis

The experimental values were analyzed by One-way ANOVA procedure of IBM SPSS Statistics ver. 22.0 (IBM SPSS Statistics, 2013, IBM, Armonk, NY, USA). The difference of means was tested by Tukey test. The effect of dietary astaxanthin supplementation levels was determined using orthogonal polynomials for linear and quadratic effects. A significant difference was expressed either *p* < 0.01 or *p* < 0.05, however *p* values 0.05 to 0.1 were sometimes given to indicate if the value tended to differ.

## 3. Results

### 3.1. Antioxidant Enzymes

There was a linear response to the addition of astaxanthin from 0 to 80 mg/kg diet, with the lowest MDA (*p* = 0.023) observed in the plasma of chicks fed 80 mg/kg astaxanthin (Table 2). Catalase (Linear, *p* < 0.01; quadratic, *p* < 0.05) and SOD (Linear, *p* < 0.05) levels in the plasma were increased by increasing astaxanthin doses in the diet, while there were no differences between treatments for the concentration of GPx, MPO, and TAC in the plasma at any dietary astaxanthin levels. Increasing dietary astaxanthin resulted in a linear decrease (*p* < 0.01) in MDA in the leg muscle. There was no significant linear or quadratic effects of dietary astaxanthin on the concentration of glutathione, SOD, catalase, and MPO in the leg muscle. There was a linear increase (*p* < 0.01) in the level of TAC in the leg muscle with increased levels of astaxanthin.

### 3.2. DPPH Radical Scavenging Activity and ABTS^+^ Reducing Activity

**Figure 1:**
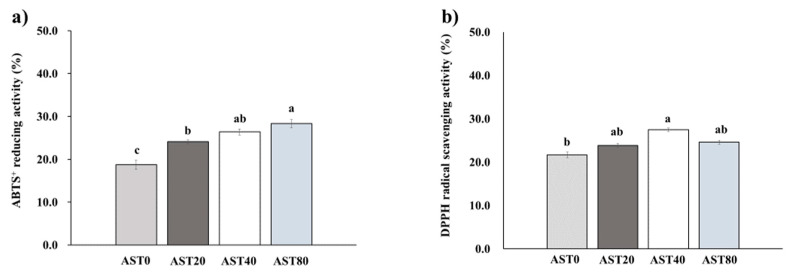
Effect of dietary astaxanthin supplementation levels on 3-ethylbenzothiazoline-6-sulfonate (ABTS^+)^ reducing activity (**a**) and 2,2-diphenyl-1-picrylhydrazyl (DPPH) radical scavenging activity (**b**) of leg muscles in broilers under high temperature. AST0, basal diet; AST20, basal diet + 20 ppm astaxanthin; AST40, basal diet + 40 ppm astaxanthin; AST80, basal diet + 80ppm astaxanthin. ABTS^+^: linear, *p* < 0.001; quadratic, *p* = 0.066. DPPH: linear, *p* = 0.048; quadratic, *p* = 0.072. Bars with different letters (a–c) differ significantly across all treatments (*p* < 0.05).

The leg muscle antioxidant capacity data showed that ABTS reducing activity was significantly increased in the AST80 treatment compared with the AST0 and AST20 treatments (Figure 1). There was no difference in ABTS reducing activity between AST80 and AST40 treatments. The AST40 treatment showed a significant increase (*p* < 0.05) in DPPH radical scavenging capacity of leg muscles compared with the AST0 treatment, however, there was no difference among AST-supplemented treatments.

### 3.3. Growth Performance, Carcass Quality, Immune Organ Ratio, Meat Color and Meat Quality

**Figure 2:**
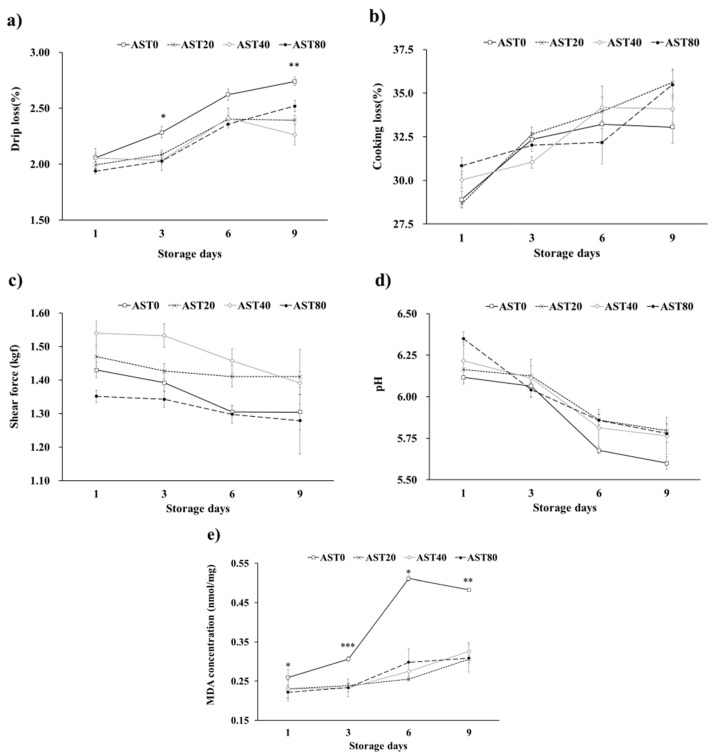
Effect of dietary astaxanthin supplementation levels on drip loss (**a**), cooking loss (**b**), shear force (**c**), pH (**d**), and malondialdehyde (MDA) concentration (**e**) in the leg muscle of broilers at 4 ℃. AST0, basal diet; AST20, basal diet + 20ppm astaxanthin; AST40, basal diet + 40ppm astaxanthin; AST80, basal diet + 80ppm astaxanthin; *, *p* < 0.05; ** *p* < 0.01; ***, *p* < 0.001.

Table 3 shows the effect of diets on growth performance, carcass characteristics, and relative immune organ weight. The effect of astaxanthin on growth performance of broilers was quadratic (*p* < 0.01) as the bodyweight gain was increased by 5.7% and 3.6% in broilers fed 20 and 40 ppm astaxanthin, respectively. There was no difference in feed intake and feed conversion ratio among the treatments. The carcass rate showed a positive linear response (*p* < 0.05) with increasing dietary astaxanthin, however, the breast muscle and drumstick rations were not affected by the treatments. A lower (*p* < 0.01) abdominal fat was detected as the dietary concentration of astaxanthin increased. The relative weight of the liver was lower (*p* < 0.01) as astaxanthin doses used in the feed increased. There were no differences in the relative organ weight of spleen and bursa Fabricius between the supplemented diets. Table 3 shows the effect of diets on breast meat color of broiler chickens. Breast meat redness (*p* < 0.05) and yellowness (*p* < 0.01) showed a linear response to increasing dietary astaxanthin levels. Although, in contrast to redness and yellowness, there was no difference in the meat lightness between the supplemented diets. The meat quality results are summarized in Figure 2. The highest drip loss of leg muscle was detected for AST0 at 3 days (2.24 ± 5.1%; *p* < 0.05) and 9 days (2.71 ± 3.6%; *p* < 0.01) of storage. Although the values differed slightly, there were no changes in cooking loss, shear force, and pH of leg muscle among the treatments during 9 days of storage. The highest MDA concentration was shown for AST0 treatment over 9 days of storage.

### 3.4. Hepatic Gene Expression and Feather Corticosterone

**Figure 3:**
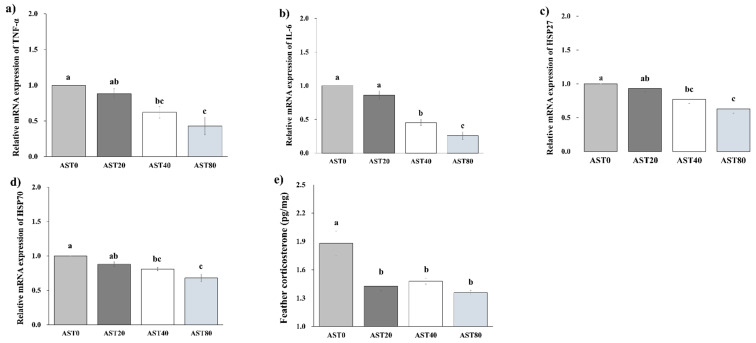
Relative mRNA expression of TNFα (**a**), IL-6 (**b**), HSP27 (**c**), and HSP70 (**d**) in the liver, and feather corticosterone concentration of broiler under high temperature (**e**). AST0, basal diet; AST20, basal diet + 20 ppm astaxanthin; AST40, basal diet + 40 ppm astaxanthin; AST80, basal diet + 80 ppm astaxanthin; TNF, Tumor necrosis factor; IL, Interleukin; HSP, Heat shock protein. Bars with different letters (a–c) differ significantly across all treatments (*p* < 0.05).

The effects of dietary astaxanthin on gene expression of immunity, stress, and antioxidant factors are shown in Figure 3. Results indicated that compared to the AST0 group, added 40 or 80 mg/kg astaxanthin significantly decreased (*p* < 0.05) TNF-α and IL-6 expression in the livers. Dietary supplemented 40 or 80 mg/kg astaxanthin significantly decreased (*p* < 0.050) the gene expression of HSP27 and HSP70 in the liver of broiler chicks as compared to the AST0 group. The feather corticosterone was significantly higher (*p* < 0.05) in the AST0 treatment compared with the AST20, AST40, and AST80 treatments.

## 4. Discussion

The antioxidative enzyme system including SOD, catalase, and GPx is the first line of defense against inflammation. It has been reported that the unique structure of astaxanthin can restore SOD levels and decreases MDA through scavenging the reactive oxygen near the surface of membrane by their polar ring, as well as inhibiting the radical reactions into the membrane by the polyene chain [29]. Alteration of the aforementioned enzyme’s activity can improve the balance between the antioxidant system and production of reactive oxygen species (ROS). Superoxide dismutase is an enzyme that protects cells against reactive oxygen species [10,11,30]. In this study, increased activity of SOD and reduced concentration of MDA in the plasma of chickens treated with astaxanthin may be associated with the increase in the activity of SOD and catalase, which scavenge lipid peroxides and hydroperoxides. The activities of catalase and SOD were decreased in the plasma of broiler chicks fed AST0, which showed that the potential of scavenging free radicals was impaired by heat stress without astaxanthin supplementation. It has been known that the production of SOD and catalase compromises during heat stress, which results in increasing MDA and oxidative stress [31]. High MDA levels have been known as an indicator of lipid peroxidation [32]. Meanwhile, the decreased lipid peroxidation level of the leg muscle by reduction of MDA activities in the astaxanthin groups was in line with increasing the redness of muscle. Although decreased MDA concentrations seem to be consistent in the plasma and leg muscle, this consistency is missing for catalase and SOD activity. These results are in agreement with the findings of other researchers studying antioxidant factors concerning the decrease of risk factors including MDA in animals treated with astaxanthin [8,15,33,34]. Although the linear decreases in the MDA concentrations in the plasma and leg muscles were in line with the TAC in the leg muscle and respective plasma enzymes such as catalase and SOD concentrations change, the detected TAC changes in plasma did not match with the current results. There might be a relationship between the accumulation of astaxanthin in storage organs such as meat and antioxidant activity rather than plasma.

Although astaxanthin has a potent antioxidant activity [15,34,35,36], previous studies did not focus on the influences of supplemental astaxanthin on the meat quality cascade related to high ambient temperatures in the broiler chickens at the total antioxidant capacity level. Therefore, this dose-dependent study was performed to evaluate the effects of astaxanthin on antioxidant status in meat, plasma, and their relationship with meat quality and color. However, the effects of astaxanthin on the scavenging capacity against DPPH and ABTS in broiler meat have not been studied, a previous study showed that the DPPH or ABTS radical-scavenging activity of astaxanthin was 72 or 220 times greater than ascorbic acid, as an antioxidant factor, in shrimp shell [37]. The enhanced ABTS and DPPH scavenging capacity due to astaxanthin supplementation was accompanied by an increase in tissue antioxidant capacity measured by TAC in the leg muscle, implicating the decrease of oxidative tissue damage in the presence of astaxanthin. In addition, elevated plasma SOD, as well as reduced MDA demonstrate the reduction of oxidative stress by astaxanthin. Therefore, our study showed that 40 or 80 mg/kg of astaxanthin are suitable concentrations used to increase the ABTS reducing and DPPH scavenging activities.

The growth performance of broiler chicks in the current study was 21.5%, 17.0%, 18.6%, and 20.7% lower than Aviagen [18] recommendation in AST0, AST20, AST40, and AST80, respectively. Moreover, feed intake was 12.1%, 9.6%, 10.1%, and 14.5% decreased in AST0, AST20, AST40, and AST80, respectively. These reductions in growth performance and feed intake may be the consequence of heat stress in this study. The behavior on oxidation of prolonged storage time of meat, which is the main factor of meat quality, helps to explain the abovementioned results. The high polyunsaturated fatty acids content increases the sensitivity of broilers meat to oxidative rancidity. During the storage period, the acidity of meats did not alter by astaxanthin concentrations. Similar results have been reported by other studies [34]. The significantly lower drip loss in astaxanthin-supplemented treatments on days 3 and 9 was in agreement with Perenlei et al. [35] who reported a lower drip loss of breast meat samples with supplementation of astaxanthin. However, the low levels of astaxanthin supplementation (15 and 30 mg/kg) did not affect drip loss of chicken breast at 1, 3, and 5 days of storage [34]. The protection of enzymes and phospholipid membranes from free radical damage seems necessary to decrease the adverse effects of heat stress. Antioxidant properties of carotenoids and natural pigments are extensively discussed in the literature [8,10,38,39]. Ao and Kim [34] also confirmed that as the levels of astaxanthin increased in the diet, the MDA concentration of meat was reduced. The main mode of action of astaxanthin antioxidant activity is associated with reactions with active radicals including hydroxyl and peroxyl radicals, which can protect lipids from oxidation through chelating free radicals [40]. The result of this study shows that astaxanthin is an effective antioxidant that protects fatty acids oxidation by controlling the body’s catalase and SOD. The meat color is reported to be an effective parameter for consumers evaluation of meat products [4,7,17]. In the current study, there was a linear difference in meat color between treated chicks and control chicks. This may be associated with the increased availability of pigments due to the presence of astaxanthin. These results are in agreement with a series of studies, which reported that dietary pigments increase the redness or yellowness of meat in Pekin ducks [34] and broiler chickens [33,35,41]. Supplementation of astaxanthin has been shown as an effective way to increase the concentration of total carotenoid in the liver, breast, and thigh of broiler chickens [2]. However, this is in contrast with the reports of [36], who noted that dietary supplementation of astaxanthin did not increase meat redness in broiler chickens. Improvement in antioxidant capacity by dietary astaxanthin supplementation may result in increased meat quality during heat stress.

The welfare and growth of animals are adversely under influence of heat stress, which causes economic losses by increasing mortality and reducing feed intake [3,10]. Corticosterone can be an indicator of chronic stress and will be increased during stress [5]. Previous reports confirmed that feather corticosterone of broiler chickens increases during long-term heat stress [42]. Consistently, our study indicated that the supplementation of astaxanthin in the diet during heat stress led to the reduction of corticosterone in the feather. Under heat stress conditions, the excessive generation of ROS damages cell integrity through the degradation of cytoskeletal proteins and peroxidation of lipids [8,9,11]. The current study showed that dietary astaxanthin supplementation reduced gene expression of HSP27 and HSP70 in the livers of broiler chicks, which may reflect the anti-stress effects of astaxanthin during the heat stress condition. The HSP70 is known as a cellular thermometer [43]. As heat stress increased, the gene expression of HSP70 was up-regulated to protect the cell damages by triggering protein expression [6,44]. The HSPs isoforms response differently to heat stress [10]. They are important biomarkers in stabilizing cytoskeletal proteins by modulation of oxidative stress and apoptotic activity during a stressful period [45]. The lower gene expression of HSPs in astaxanthin-supplemented treatments may be due to the antioxidant effects of astaxanthin that reduced the adverse influences of heat stress. In addition, cytokines are the predominant mediators of inflammation with an essential role in mediating the inflammatory response during heat stress in living organisms [38]. Several studies have confirmed the relationship between the inflammatory response of the immune organs and the expression of inflammatory cytokines [5]. The most effective role played by TNF-α, as the most important cytokine, is related to its potent cytoprotective and antioxidant activity in response to injuries [5,46], and activating lymphocytes and neutrophils to produce the other cytokines [47]. The TNF-α was down-regulated in the AST80 treatment but not in the AST40 or AST20 treatments. A previous study also showed that dietary astaxanthin levels of 15 or 30 mg/kg cannot affect the gene expression of TNF- α in the liver of Pekin ducks [34]. In addition, down-regulation of anti-inflammatory cytokine IL-6 might suppress the induced inflammation from heat stress [3].

## 5. Conclusions

In conclusion, to maintain the antioxidant level needed for protecting lipids from peroxidation, the antioxidant defense system of broiler chickens during heat stress can be enhanced by astaxanthin supplementation. Dietary astaxanthin could improve meat color and quality through its pigmentation and antioxidant effects in heat-stressed broilers. Moreover, lower cytokines were produced in chickens fed astaxanthin. This, therefore, confirms that astaxanthin is an effective additive to enhance the meat quality, growth performance, and immune status of broiler chickens during heat stress conditions.

## Figures and Tables

**Table 1:** Primer sequences used in real-time PCR.

Genes	Primer Sequences (5′–3′)	Annealing Temp. (°C)	Product Size (bp)	Gene Accession Number	References
*β-actin*	F: CAACACAGTGCTGTCTGGTGGTA	58	205	X00182	[25]
	R: ATCGTACTCCTGCTTGCTGATCC				
*TNF-α*	F: AGATGGGAAGGGAATGAACC	60	164	NM_204267.1	[26]
	R: GACGTGTCACGATCATCTGG				
*IL-6*	F: CGTGTGCGAGAACAGCATGGAGA	60	110	NM_204628.1	[25]
	R: TCAGGCATTTCTCCTCGTCGAAGC				
*HSP27*	F: ACACGAGGAGAAACAGGATGAG	60	158	NM_205290.1	[26]
	R: ACTGGATGGCTGGCTTGG				
*HSP70*	F: GGTAAGCACAAGCGTGACAATGCT	55	116	AY143693.1	[26]
	R: TCAATCTCAATGCTGGCTTGCGTG				

TNF, Tumor necrosis factor; IL, Interleukin; HSP, Heat shock protein; GPX, glutathione peroxidase.

**Table 2:** Effect of dietary astaxanthin supplementation on oxidation statues in plasma and muscle of broiler under hot temperature.

Items	Dietary Astaxanthin Supplementation (ppm)				SEM	*p*-Values	
	0	20	40	80		Linear	Quadratic
Plasma (35 d)							
Malodialdehyde (nmol/mL)	16.38	14.08	11.37	9.50	0.896	0.023	0.889
Glutathione (μmol/mL)	5.73	4.99	6.28	5.61	0.196	0.572	0.932
Superoxide dismutase (U/mL)	50.63	54.77	62.10	78.08	3.641	0.027	0.354
Catalase (nmol/min/mL)	0.449	0.532	0.553	0.651	0.019	0.001	0.776
Myeloperoxidase (U/mL)	28.60	26.41	25.36	25.88	0.968	0.684	0.507
Total antioxidant capacity (U/mL)	0.563	0.593	0.607	0.650	0.212	0.185	0.880
Leg muscle (35 d)							
Malodialdehyde (nmol/mg)	0.259	0.230	0.230	0.221	0.005	0.003	0.184
Glutathione (μmol/mg)	2.18	2.33	2.54	2.89	0.762	0.111	0.756
Superoxide dismutase (U/mg)	33.61	36.47	33.17	36.45	1.040	0.588	0.920
Catalase (nmol/min/mg)	0.604	0.644	0.762	0.653	0.027	0.284	0.175
Myeloperoxidase (U/mL)	3.88	2.74	3.26	3.73	0.234	0.971	0.097
Total antioxidant capacity (U/mg)	0.044	0.047	0.050	0.062	0.002	0.004	0.284

SEM, standard error of means.

**Table 3:** Effect of dietary astaxanthin supplementation on carcass characteristics, relative weights of organs, and meat color of broiler under hot temperature.

Items	Dietary Astaxanthin Supplementation (ppm)				SEM	*p*-Values	
	0	20	40	80		Linear	Quadratic
Growth performance (22–35 d)							
Body weight gain (g/bird)	1066	1127	1105	1076	9.38	0.858	0.001
Feed intake (g/bird)	1981	2037	2026	1927	59.59	0.524	0.209
Feed conversion ratio	1.86	1.81	1.83	1.79	0.05	0.426	0.957
Carcass characteristics (35 d)							
Carcass rate (%)	69.55	70.70	71.13	71.49	0.326	0.035	0.536
Breast muscle (g/100 g BW)	11.96	10.91	10.59	10.78	0.368	0.265	0.419
Drumsticks (g/100 g BW)	10.33	10.08	10.29	9.60	0.244	0.388	0.667
Abdominal fat (g/100 g BW)	1.02	0.72	0.76	0.61	0.051	0.005	0.430
Relative weights of organs (35 d)							
Liver (g/100 g BW)	2.59	2.50	2.50	2.37	0.031	0.016	0.748
Spleen (g/100 g BW)	0.082	0.078	0.088	0.087	0.004	0.543	0.927
bursa of Fabricius (g/100 g BW)	0.088	0.093	0.098	0.100	0.002	0.074	0.729
Meat color (35 d)							
Lightness (L*)	47.49	46.31	45.83	45.90	2.09	0.580	0.766
Redness (a*)	10.51	12.90	13.74	13.81	1.04	0.032	0.277
Yellowness (b*)	8.47	9.08	10.82	12.47	0.67	0.001	0.445

SEM, standard error of means; BW, body weight.
